# Impact of Fluoxetine on Behavioral Invigoration of Appetitive and Aversively Motivated Responses: Interaction With Dopamine Depletion

**DOI:** 10.3389/fnbeh.2021.700182

**Published:** 2021-07-09

**Authors:** Carla Carratalá-Ros, Laura López-Cruz, Andrea Martínez-Verdú, Régulo Olivares-García, John D. Salamone, Mercè Correa

**Affiliations:** ^1^Àrea de Psicobiologia, Universitat Jaume I, Castelló, Spain; ^2^Behavioral Neuroscience Division, University of Connecticut, Storrs, CT, United States

**Keywords:** dopamine, fluoxetine, serotonin, motivation, effort, vigor, depression

## Abstract

Impaired behavioral activation and effort-related motivational dysfunctions like fatigue and anergia are debilitating treatment-resistant symptoms of depression. Depressed people show a bias towards the selection of low effort activities. To determine if the broadly used antidepressant fluoxetine can improve behavioral activation and reverse dopamine (DA) depletion-induced anergia, male CD1 mice were evaluated for vigorous escape behaviors in an aversive context (forced swim test, FST), and also with an exercise preference choice task [running wheel (RW)-T-maze choice task]. In the FST, fluoxetine increased active behaviors (swimming, climbing) while reducing passive ones (immobility). However, fluoxetine was not effective at reducing anergia induced by the DA-depleting agent tetrabenazine, further decreasing vigorous climbing and increasing immobility. In the T-maze, fluoxetine alone produced the same pattern of effects as tetrabenazine. Moreover, fluoxetine did not reverse tetrabenazine-induced suppression of RW time but it reduced sucrose intake duration. This pattern of effects produced by fluoxetine in DA-depleted mice was dissimilar from devaluing food reinforcement by pre-feeding or making the food bitter since in both cases sucrose intake time was reduced but animals compensated by increasing time in the RW. Thus, fluoxetine improved escape in an aversive context but decreased relative preference for active reinforcement. Moreover, fluoxetine did not reverse the anergic effects of DA depletion. These results have implications for the use of fluoxetine for treating motivational symptoms such as anergia in depressed patients.

## Introduction

Motivational symptoms such as psychomotor retardation, anergia, lack of energy, lassitude, fatigue, and reduced exertion of effort are common and critical in major depressive disorder (Stahl, 2002; Demyttenaere et al., 2005; Salamone et al., 2007; Treadway and Zald, 2011). These highly debilitating symptoms in depression are strongly correlated with problems with social function and employment (Tylee, 1999; Stahl, 2002, 2017; Hodgetts et al., 2017). They are also highly resistant to treatment and often remain as residual symptoms after remission (Stahl, 2002; Nutt et al., 2007; Fava et al., 2014). Many common antidepressants including serotonin (5-HT) transporter (SERT) inhibitors such as fluoxetine and citalopram are useful for treating mood symptoms in depression (Rosenblau et al., 2012; Rizvi et al., 2013; Hieronymus et al., 2016), but they are relatively ineffective for treating motivational dysfunctions, and, in fact, it has been suggested that SERT inhibitors may exacerbate or induce these symptoms in some patients (Nutt et al., 2007; Targum and Fava, 2011; Padala et al., 2012; Stenman and Lilja, 2013; Fava et al., 2014; Rothschild et al., 2014). However, systematic studies are still lacking.

Because of the clinical significance of motivational dysfunctions, it is critical to develop animal models that allow researchers to study a broad range of impairments, and also to assess the ability of different drugs to reverse them. Thus, some studies have focused upon different tasks involving effort-based decision-making that offer rodents the possibility of choosing between high-effort instrumental actions leading to more valued reinforcers or to choose the low-effort options leading to less valued reinforcers. Conditions associated with depression, including stress (Shafiei et al., 2012; Bryce and Floresco, 2016, 2019) and pro-inflammatory cytokines (Nunes et al., 2014; Goldsmith et al., 2016; Yohn et al., 2016a), as well as dopamine (DA) receptor antagonism (Pardo et al., 2012, 2015; Yohn et al., 2015a; Correa et al., 2016; Yang et al., 2020) or DA depletion (Nunes et al., 2013; Randall et al., 2014; Yohn et al., 2015b; López-Cruz et al., 2018; Rotolo et al., 2019; Carratalá-Ros et al., 2020) can affect effort-related decision-making and bias animals towards the low effort options. These results obtained from animal studies are consistent with clinical data showing a reduced selection of high effort alternatives in depressed people tested on tasks of effort-based choice (Treadway et al., 2012; Chong et al., 2016). Administration of the vesicular monoamine transporter (VMAT-2) inhibitor tetrabenazine (TBZ), which blocks the monoamine storage and leads to a striatal DA depletion at low doses in primates (Pettibone et al., 1984), rats (Nunes et al., 2013; Podurgiel et al., 2015, 2016; Yohn et al., 2015b), and mice (López-Cruz et al., 2018; Carratalá-Ros et al., 2020) has been used to induce effort-related motivational impairments in rodents (Nunes et al., 2014; Yohn et al., 2015b, 2016b; Correa et al., 2018; López-Cruz et al., 2018; Rotolo et al., 2019; Carratalá-Ros et al., 2020). This drug is used in the treatment of Huntington’s disease and has been reported to produce depressive symptoms including fatigue in people (Frank, 2009; Guay, 2010). Recently, we have also demonstrated that TBZ induces depressive symptoms in the forced swim test (FST); mice show less vigorous scaping behaviors and increase immobility (Carratalá-Ros et al., 2020).

Willingness to do effort can also be studied in a recreational context in which there is no stress from which to escape, and no work required to obtain a meal. Thus, voluntary vigorous wheel running was used in a recently developed T-maze choice task as the high effort/highly preferred option (Correa et al., 2016; López-Cruz et al., 2018; Carratalá-Ros et al., 2020). This paradigm assesses the impact of drugs, homeostatic or environmental manipulations on behavioral activation, and effort-related choice. This task allows the animal to freely choose between running on a running wheel (RW), consuming sucrose pellets, or sniffing a neutral non-social odor. It has been demonstrated that TBZ produced a dose-dependent shift in response selection, reducing preference for the energy-requiring reinforcer (wheel running), but concurrently increasing time with the second preferred reinforcer (palatable food) that required little effort to obtain (López-Cruz et al., 2018; Carratalá-Ros et al., 2020). Thus, animals do not show anhedonia towards the food but show an anergic behavioral pattern. This is also a model of voluntary physical activity. It has been observed that a lack of activity can contribute to the development of depression (Lambert, 2006), and in depressed people, symptoms such as loss of interest, motivation and energy, and generalized fatigue problems interfere with participation in exercise (Knapen et al., 2015), especially because the choice to engage in voluntary physical activity is always undertaken in relation to the possible selection of other more sedentary alternatives.

The ability of antidepressants that are monoamine uptake inhibitors to reverse the effects of TBZ on effort-based choice in rodents differs depending upon their specific mechanism of action. For example, SERT inhibitors such as fluoxetine or s-citalopram failed to reverse the low-effort bias induced by TBZ in rats tested in operant paradigms in which animals had to work (lever press) to get access to more palatable food or could choose to approach and consume a less preferred but freely available chow (Yohn et al., 2016b,c,e). Moreover, fluoxetine either acutely or chronically administered had the same impact on effort-based choice (Yohn et al., 2016b,c,e). These behavioral effects of fluoxetine were paralleled by a reduction in nucleus accumbens (Nacb) core DA levels (Yohn et al., 2016b,c,e). However, DA transport (DAT) inhibitors reverse TBZ effects on effort-based-choice and increased DA in Nacb (Nunes et al., 2013; Randall et al., 2014; Yohn et al., 2016b,c,d). On the contrary, in the FST fluoxetine effectively enhances active behaviors and reduces passive ones (Petit-Demouliere et al., 2005; Jang et al., 2009), the opposite pattern to TBZ (Carratalá-Ros et al., 2020).

Since the effects of fluoxetine on activational aspects of motivation can be seen after acute administration, the present study explored the effect of fluoxetine administered acutely, alone or in combination with the DA depleting agent TBZ, in a non-stressful context (the 3-choice RW T-maze task) that evaluates relative preference for active reinforcers vs. more sedentary options, and compared those results with actions in the FST, which involves a stressful test setting and measures vigorous scaping vs. passive responses. We also evaluated these drugs and their combination on measures of anxiety (dark and light box, DL box and the elevated plus maze, EPM) as a potential explanation for their effects in those motivational paradigms. We also compared the effects of fluoxetine with the effect of behavioral manipulations that change the emotional or homeostatic value of food on the performance of the RW T-maze task.

The proposed work is not presenting a global model of depression *per se*, or providing a general screening of antidepressant drugs, but rather focused on a specific behavioral component (active exertion of physical effort) that is particularly important for the motivational symptoms of depression, and potentially other disorders as well.

## Materials and Methods

### Animals

CD1 adult male mice (*N* = 106) purchased from Janvier, France S.A. were 8–14 weeks old (40–50 g) at the beginning of the study. Mice were housed in groups of three or four per cage, with standard laboratory rodent chow and tap water available *ad libitum*. The colony was kept at a temperature of 22 ± 2°C with lights on from 08:00 h to 20:00 h. All animals were under a protocol approved by the Institutional Animal Care and Use Committee of Universitat Jaume I. All experimental procedures complied with directive 2010/63/EU of the European Parliament and of the Council, and with the “Guidelines for the Care and Use of Mammals in Neuroscience and Behavioral Research,” National Research Council 2003, USA. All efforts were made to minimize animal suffering and to reduce the number of animals used.

### Pharmacological Agents

Fluoxetine (CIMYT Quimica SL, Spain) was dissolved in 0.9% saline and administered 30 min before testing. The range of doses of fluoxetine was based on studies involving classical mice antidepressant screening tests (Lucki et al., 2001) and effort-related behavioral tests (Yohn et al., 2016b, c). In order to conduct the reversal behavioral experiments, the VMAT-2 blocker tetrabenazine (TBZ; CIMYT Quimica SL, Spain) was used. TBZ was dissolved in a vehicle solution of 0.9% saline (80%) plus dimethylsulfoxide (DMSO 20%, final pH 5.5) and administered 120 min before testing. Time elapsed after injection and the dose of 8.0 mg/kg TBZ were selected based on previous behavioral work (Correa et al., 2018; López-Cruz et al., 2018; Carratalá-Ros et al., 2020) and neurochemical studies (López-Cruz et al., 2018) demonstrating that in mice this is an optimal dose and time lead to deplete DA. DMSO (20% v/v) and saline were used as the control group. All the substances were administered intraperitoneally (IP).

### Testing Procedures

All behavioral procedures started 2 h after the light period started. The behavioral test room was illuminated with a soft light, and external noise was attenuated.

#### Forced Swim Test (FST)

This paradigm is considered to be a model of behavioral despair and is used as a test for assessing depressive-like states and for evaluating drugs with potential as antidepressants (Porsolt et al., 1977). Immobility was defined as the animal remaining motionless, making only minor movements to balance the body and keep the head above the water. Mild swimming was recorded when animals carried out horizontal movements with their forepaws, leading to the displacement of the body throughout the swim chamber (Armario et al., 1988). In addition, we also assessed escape-related mobility such as climbing (Armario et al., 1988). Climbing is defined as any energetic and vertical movement of all four limbs against the wall of the tank. Naïve mice were placed in a transparent cylindrical glass tank (26 cm high and 18 cm diameter) filled with water (14 cm) and maintained at a temperature of 25°C. Water was changed between animals. During the 6-min test, mice were videotaped from the side, and climbing, immobility, and swimming were later measured by an observer unaware of the experimental condition. After the test, mice were dried with a soft towel, put back in a box with absorbing paper under a warming light, and were monitored for 10 min.

#### Dark and Light Box (DL)

The DL test is based on the conflict between the tendency to explore a novel environment and the avoidance of a brightly lit open area (Blumstein and Crawley, 1983). The DL apparatus consisted of a polypropylene chamber divided into two compartments by a partition containing a small opening (5 cm H × 5 cm W). The light compartment (25 cm W × 25 cm H × 25 cm L) was open, painted in white, and illuminated (335 lx), while the dark compartment (25 cm W × 25 cm H × 18 cm L) was painted in black and had a removable ceiling to close it (Kulesskaya and Voikar, 2014). To start the test session, mice were individually placed in the dark chamber facing one corner. Test sessions were videotaped, and the total number of crosses and the total time spent in the lit chamber were recorded for 5 min (López-Cruz et al., 2014; Carratalá-Ros et al., 2020).

#### Elevated Plus Maze (EPM)

The EPM consists of two open and two enclosed arms (65 cm L × 5 cm W) arranged in a plus configuration and intersecting in a central platform. It is made of black polypropylene and is elevated 50 cm above the floor. The open arms have a 1 cm border around their perimeter and the closed arms have a 20 cm translucent wall. This anxiety paradigm measures the avoidance that rodents show to high open spaces. Under normal conditions, mice spend more time in and make more entries into the closed arms of the maze (Lister, 1987). Animals were placed in the central platform with their head pointing at one enclosed arm, and they were assessed for 5 min. Sessions were videotaped and a trained observer registered total time spent in the open arms, the ratio of entries into the open arms compared to total arm entries, and total entries in the four arms as an index of locomotion. An entry into an arm was recorded when the animal crossed with all four legs the line that connected that arm with the central platform (López-Cruz et al., 2014).

#### T-Maze RW-Sucrose-Odor Choice Task

The 3-choice-T-maze apparatus consisted of a central area that led to three arms (López-Cruz et al., 2018). In one of them, sucrose pellets (TestDietTM, 50% sucrose, 45 mg each) were available, in another arm there was an RW, and in the third arm, there was a hole with a cotton ball soaked with a fruity odor. Training as well as test sessions, lasted 15 min. Mice were trained in one session per day, 5 days a week. Training phase 1: to avoid neophobia to the sweet-tasting pellets, animals were enclosed in that arm with the food during five initial sessions. Training phase 2: during two more weeks animals were exposed to the T-maze with free access to the three stimuli, each one in a different arm. Test phase: This phase lasted between two or five more weeks depending on the experiment. For each week, there were four training sessions plus a testing session in which animals received drug injections or were exposed to the food manipulations. The day before the food manipulation was considered as the baseline (BL). Entries into the arms of the T-maze were simultaneously recorded. All these measures were used based on previous studies (Correa et al., 2016; López-Cruz et al., 2018; Carratalá-Ros et al., 2020). Time interacting with the stimuli was selected as the main dependent measure because it allowed for the evaluation of interactions with the three different stimuli with the same units (i.e., time). Time allocation is a useful measure of preference, relative reinforcement value, and response choice (Baum and Rachlin, 1969).

### Statistical Analyses

All of the experiments were single factor designs, and thus all experiments were analyzed with either simple between groups analysis of variance (ANOVA), repeated measures ANOVA, or Student’s *t*-test. Normally distributed and homogenous data (according to Kolmogorov–Smirnov test) for the FST, DL, and EPM, employed between groups design and data were analyzed by one-way ANOVA. Normally distributed data in the T-maze experiment followed a within groups design. Thus, when more than two experimental conditions were used, such as in the fluoxetine or the TBZ plus fluoxetine experiments, data were analyzed by repeated measures ANOVA. When the overall ANOVA was significant, non-orthogonal planned comparisons using the overall error term were used to compare each treatment with the vehicle control group (Keppel, 1991). For these comparisons, α level was kept at 0.05 because the number of comparisons was restricted to the number of treatments minus one. The effects of changing the taste of the pellets and pre-feeding animals were evaluated by Student’s *t*-test for dependent samples. All data were expressed as mean ± SEM, and significance was set at *p* < 0.05. STATISTICA 7 software was used.

## Results

### Experiment 1

Effect of fluoxetine in the FST. Different groups of animals (*N* = 31) received one dose of fluoxetine (vehicle, 10.0, 15.0, or 20.0 mg/kg) and 30 min after the injection were placed in the FST for 6 min. Mice were exposed only once to the FST since behavioral habituation develops in one session.

**Figure 1:**
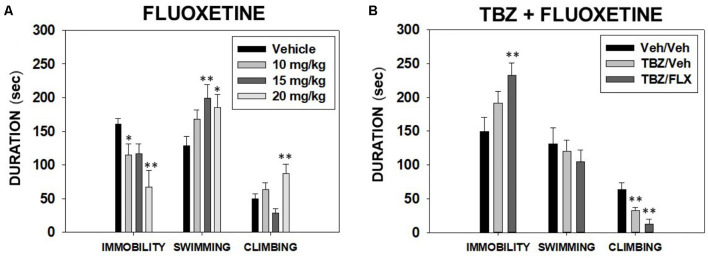
Effect of fluoxetine (Vehicle, 10, 15 or 20 mg/kg) **(A)** and tetrabenazine (TBZ) plus fluoxetine combination (Veh + Veh, TBZ + Veh, or TBZ + Fluoxetine 20 mg/kg **(B)** on the duration of immobility, swimming, and climbing behavior in the forced swim test (FST) assessed for 6 min. Groups were analyzed using one-way analysis of variance (ANOVA) for independent samples in each dependent variable. Bars represent mean ± SEM of accumulated seconds. *p < 0.05, **p < 0.01 significantly different from the control group.

The one-way ANOVA showed a significant effect on immobility time (*F*_(3,27)_ = 5.0, *p* < 0.01), time swimming (*F*_(3,27)_ = 3.41, *p* < 0.05), and time climbing (*F*_(3,27)_ = 5.68, *p* < 0.01). Planned comparisons revealed that the groups that received 10.0 and 20.0 mg/kg displayed significantly less time to be immobile (*p* < 0.05, *p* < 0.01 respectively) in comparison with the vehicle group. The two highest doses of fluoxetine (15.0 and 20.0 mg/kg) produced an increase in time spent swimming (*p* < 0.01 and *p* < 0.05 respectively), and only 20.0 mg/kg of fluoxetine increased time climbing in the FST (*p* < 0.01) in comparison with the vehicle group (Figure 1A).

### Experiment 2

Effect of TBZ and fluoxetine combination in the FST. Three different groups of naïve mice (*N* = 38) received a combination of TBZ (8.0 mg/kg, a dose that was shown to be effective in the FST; Carratalá-Ros et al., 2020) plus vehicle or the highest dose of fluoxetine (20.0 mg/kg) or the combination of both vehicles. Fluoxetine was given 90 min after TBZ and 30 min before animals were placed in the FST.

The one-way ANOVA for time spent swimming (*F*_(2,35)_ = 0.34; *p* = 0.70) was not significant. However, the ANOVAs for immobility and climbing were significant (*F*_(2,35)_ = 3.76, *p* < 0.05; *F*_(2,35)_ = 9.10, *p* < 0.01 respectively). Planned comparisons revealed that the group that received TBZ 8.0 mg/kg plus vehicle, and also the group that received TBZ 8.0 + fluoxetine 20.0 mg/kg displayed significantly less climbing than the vehicle group (*p* < 0.01). Finally, TBZ+ fluoxetine at 20.0 mg/kg increased the time of immobility compared with the vehicle group (*p* < 0.01; Figure 1B).

### Experiment 3

Effect of fluoxetine in the DL and EPM paradigms. Independent groups of mice (*N* = 35) received one dose of fluoxetine (vehicle, 10.0, 15.0, or 20.0 mg/kg) and 30 min after injection were first placed in the DL box for 5 min. Immediately after this test, they were placed in the EPM for five more minutes. Mice were exposed only once to both paradigms since behavioral habituation develops in one session.

**Figure 2:**
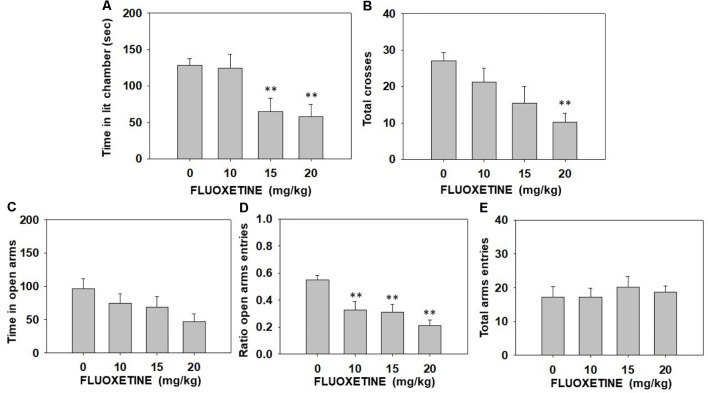
Effect of fluoxetine (Vehicle, 10, 15 or 20 mg/kg) on time spent in the lit chamber **(A)** and the total of entries between the two compartments **(B)** of the DL box, and on time spent in the open arms **(C)**, ratio to the open arms **(D)**, and the total of entries to the four arms **(E)** of the EPM during 5 min each test. Groups were analyzed using one-way ANOVA for independent samples in each dependent variable. Bars represent mean ± SEM of accumulated seconds. **p < 0.01 significantly different from the vehicle group. Abbreviations: DL box, dark and light box; EPM, elevated plus maze.

The one-way ANOVA showed a significant effect on time spent in the illuminated arena (*F*_(3,31)_ = 5.11, *p* < 0.01) of the DL box. Moreover, the one-way ANOVA also revealed a significant effect on the total number of entries into both compartments of the DL box (*F*_(3,31)_ = 2.99, *p* < 0.05). Planned comparisons indicated that mice treated with fluoxetine at 15.0 and at 20.0 mg/kg spent less time in the lit chamber (*p* < 0.01) compared to the vehicle group. Moreover, the highest dose of fluoxetine produced a decrease in the total number of entries into both compartments (*p* < 0.01) in comparison with the vehicle group (Figures 2A,B). The one-way ANOVA for the effect of fluoxetine in the EPM showed a significant effect on the ratio of entries into the open arms (*F*_(3,31)_ = 7.49, *p* < 0.01). Planned comparisons showed that all doses of fluoxetine reduced the ratio of entries compared to vehicle (*p* < 0.01; Figure 2D). However, the one-way ANOVA did not show a significant effect on time spent in the open arms (*F*_(3,31)_ = 1.91, *p* = 0.14) or the total number of entries into all arms of the EPM (*F*_(3,31)_ = 0.25, *p* = 0.85; Figures 2C,E).

### Experiment 4

Effect of TBZ and fluoxetine combination in the DL and EPM paradigms. Three different groups of naïve mice (*N* = 32) received a combination of TBZ (8.0 mg/kg) plus vehicle or the highest dose of fluoxetine (20.0 mg/kg) or the combination of both vehicles. Fluoxetine was given 90 min after TBZ and 30 min before animals were placed in the DL box for 5 min, and immediately after this test, they were placed in the EPM for five more minutes.

**Figure 3:**
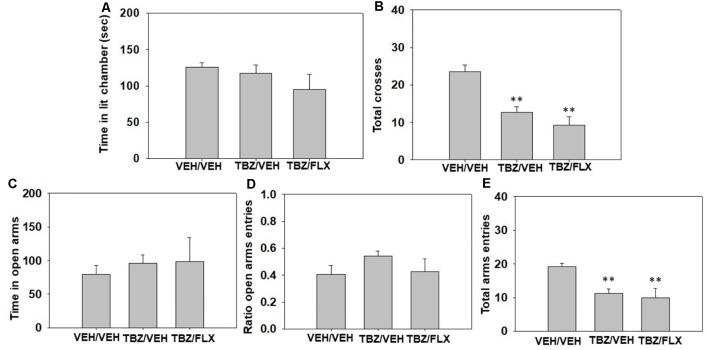
Effect of TBZ plus fluoxetine combination (Veh + Veh, TBZ + Veh, or TBZ + Fluoxetine 20 mg/kg) on time spent in the lit chamber **(A)** and the total of entries between the two compartments **(B)** of the DL box, and on time spent in the open arms **(C)**, ratio to the open arms **(D)**, and the total of entries to the four arms **(E)** of the EPM during 5 min each test. Each dependent variable was analyzed by one-way ANOVA for independent samples. Bars represent mean ± SEM of accumulated seconds. **p < 0.01 significantly different from vehicle-vehicle group.

The one-way ANOVAs did not show any significant effect of the treatment on anxiety parameters such as time spent in the lit chamber of the DL box (*F*_(2,27)_ = 1.23, *p* = 0.30), time spent in the open arms (*F*_(2,27)_ = 2.27, *p* = 0.80) or ratio of entries (*F*_(2,27)_ = 1.69, *p* = 0.20) evaluated in the EPM paradigm (Figure 3). However, different one-way ANOVAs did show significant effects on locomotion seen in the total of entries between the two compartments of the DL box (*F*_(2 27)_ = 16.93, *p* < 0.01), and the total of entries into all arms of the EPM (*F*_(2,27)_ = 6.64, *p* < 0.01). Planned comparisons revealed that the groups treated with 8.0 mg/kg of TBZ plus vehicle and the combination of TBZ plus fluoxetine both decreased the total number of entries in comparison with the vehicle group (*p* < 0.01) in the DL box (Figure 3B). The same effect was observed in the EPM: the groups treated with TBZ plus vehicle, and TBZ plus fluoxetine were different from the vehicle treated group (*p* < 0.01; Figure 3E).

### Experiment 5

Effect of fluoxetine on preference for active reinforcers in the 3-choice-T-maze task. After reaching a stable BL level of performance in the T-maze, animals (*N* = 10) received fluoxetine (vehicle, 10.0, 15.0, and 20.0 mg/kg) and, after 30 min, were placed in the T-maze for 15 min. Since BL behavior does not change, animals received one dose of the drug every week in a randomly varied order. The T-maze paradigm requires a BL performance of 2 weeks before drug tests start, and that performance was maintained across weeks, thus allowing a repeated measures design. We have observed that fluoxetine does not produce sensitization or tolerance when administered once per week.

**Figure 4:**
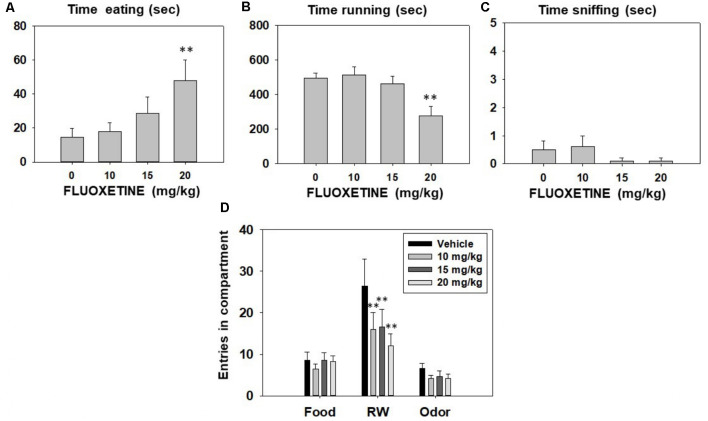
Effect of fluoxetine (Vehicle, 10, 15 and 20 mg/kg) on time eating **(A)**, time running **(B)**, time sniffing **(C)**, entries into compartments **(D)**, and in the T-maze task assessed during 15 min. Data for each variable were analyzed by repeated measures ANOVA. Bars represent mean ± SEM of accumulated seconds or number of entries. **p < 0.01 significantly different from vehicle.

Repeated measures ANOVA showed that fluoxetine did not produce any significant effect on time spent sniffing the neutral odor (*F*_(3,27)_ = 0.90, *p* = 0.45). However, fluoxetine produced a significant effect on time spent eating (*F*_(3,27)_ = 6.26, *p* < 0.01), and time spent running in the RW (*F*_(3,27)_ = 15.13, *p* < 0.01). Planned comparisons revealed that fluoxetine 20.0 mg/kg treated-mice spent significantly more time consuming sucrose pellets (*p* < 0.01), and less time running in the RW (*p* < 0.01) compared to the vehicle group (Figures 4A–C). Finally, repeated measures ANOVAs indicated that fluoxetine did not produce significant effects on the number of entries into the food compartment (*F*_(3,27)_ = 0.97, *p* = 0.42), and into the neutral odor compartment (*F*_(3,27)_ = 1.98, *p* = 0.14). However, the SERT inhibitor did significantly reduce the total number of entries into the RW compartment (*F*_(3,27)_ = 5.43, *p* < 0.01). Planned comparisons revealed that all doses of fluoxetine produced a decrease in the total of entries to the RW compartment in comparison with the vehicle group (*p* < 0.01; data shown in Figure 4D).

### Experiment 6

Effect of TBZ and fluoxetine combination on preference for active reinforcers in the 3-choice-T-maze task. The same statistical design was used. A new group of mice (*N* = 9) received TBZ (veh, 8.0 mg/kg) and, 90 min later, a dose of fluoxetine (veh, 10.0, 15.0, 20.0 mg/kg), 30 min later they were placed in the T-maze during 15 min. Animals received one drug combination (veh-veh, TBZ-veh, TBZ-10, TBZ-15 and TBZ20 mg/kg) every week during 5 weeks in a random varied order.

**Figure 5:**
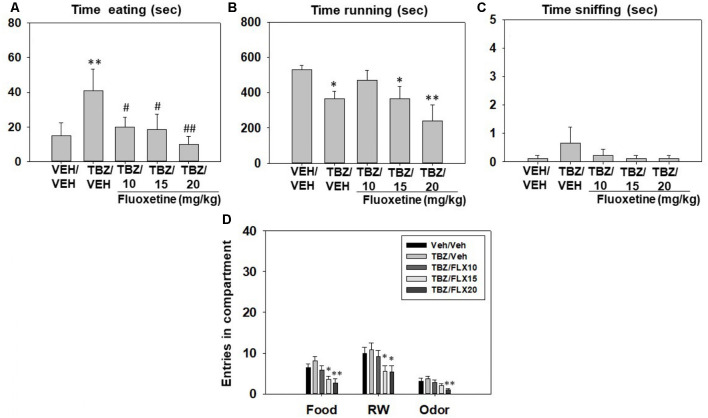
Effect of TBZ (Vehicle or 8 mg/kg) plus fluoxetine (Vehicle, 10, 15, and 20 mg/kg) combination on time eating **(A)**, time running **(B)**, time sniffing **(C)**, entries into compartments **(D)**, and in the T-maze task assessed during 15 min. Repeated measures ANOVA was used to analyze each variable. Bars represent mean ± SEM of accumulated seconds or number of entries. *p < 0.05, **p < 0.01 significantly different from Veh + Veh. ^#^*p* < 0.05, ^##^*p* < 0.01 significantly different from TBZ + Veh.

Repeated measures ANOVA showed a significant effect of treatment on time spent eating sucrose pellets (*F*_(4,32)_ = 3.33, *p* < 0.05) and time spent running in the RW (*F*_(4,32)_ = 3.86, *p* < 0.01), but no significant effect on time sniffing the neutral odor (*F*_(4,32)_ = 0.77, *p* = 0.54). Planned comparisons showed that mice treated with TBZ 8.0 mg/kg plus vehicle spent less time running (*p* < 0.05) than the vehicle-vehicle control condition. Also, fluoxetine suppressed time running in the RW in TBZ- treated mice at doses of 15.0 and 20.0 mg/kg in comparison with the vehicle-vehicle condition (*p* < 0.05 and *p* < 0.01 respectively). Planned comparisons for time consuming sucrose revealed that mice treated with TBZ plus vehicle increased time consuming sucrose pellets (*p* < 0.01) in comparison with the vehicle-vehicle group, but all doses of fluoxetine in combination with TBZ suppressed time consuming sucrose pellets (for TBZ+ 10.0 and TBZ+15.0 mg/kg *p* < 0.05 and for TBZ+20 mg/kg *p* < 0.01) in comparison with the TBZ plus vehicle condition (Figures 5A–C).

Finally, repeated measures ANOVAs showed a significant effect of treatment on the number of entries to the RW compartment (*F*_(4,32)_ = 2.89, *p* < 0.05), entries into the food compartment (*F*_(4,32)_ = 5.46, *p* < 0.01), and total entries into the odor compartment (*F*_(4,32)_ = 3.46, *p* < 0.01). Planned comparisons showed that TBZ+ fluoxetine at 15.0 and 20.0 mg/kg produced a decrease in entries into the RW compartment (*p* < 0.05), and into the food compartment (*p* < 0.05 and *p* < 0.01 respectively) compared with their respective vehicle condition. Only the highest dose of fluoxetine (20.0 mg/kg) in combination with TBZ decreased entries to the odor compartment (*p* < 0.01) in comparison with the vehicle condition (Figure 5D).

### Experiment 7

Manipulations that devalue sucrose reinforcement: change in taste with bitter pellets. Animals (*N* = 8) were trained as described before, and after reaching stable levels of interaction with the three different reinforcers, a drop of quinine (1.0 g/L) was added to the sweet pellets in order to make them bitter. BL behavior was assessed the day before the bitter pellets were substituted for the regular ones.

**Figure 6:**
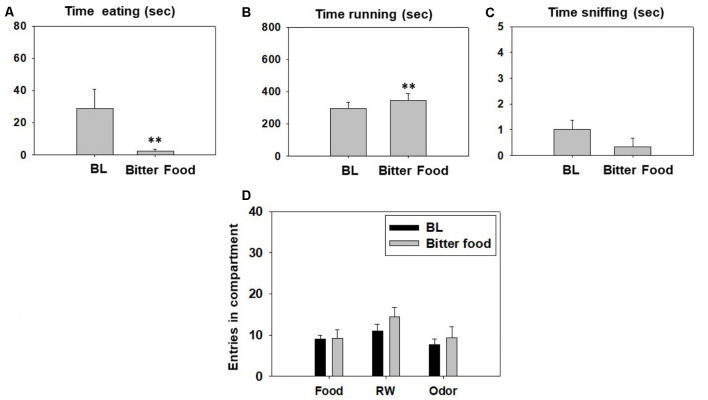
Effect of bittering the sucrose pellets on time eating **(A)**, time running **(B)**, time sniffing **(C)**, entries into compartments **(D)**, and in the T-maze task assessed during 15 min. Data were analyzed with the Student’s *t*-test for dependent samples. Bars represent mean ± SEM of accumulated seconds or number of entries. **p < 0.01 significantly different from baseline (BL) condition.

A series of Student’s *t*-tests for dependent samples for each variable showed a significant effect of bitter pellets (*t*_(5)_ = 4.56, *p* < 0.01) on time running and on time spent eating (*t*_(5)_ = −2.53, *p* < 0.01) compared with their respective BL. However, there was no significant effect of bitter food on time sniffing the neutral odor (*t* = 2.0, *p* = 0.10; Figures 6A–C). Finally, *t*-tests for dependent samples failed to demonstrate significant differences between both conditions on total entries to the food compartment (*t*_(5)_ = −0.09, *p* = 0.92), total entries to the RW compartment (*t*_(5)_ = −2.23, *p* = 0.07), and total entries to the odor compartment (*t*_(5)_ = −0.58, *p* = 0.58; Figure 6D).

### Experiment 8

Manipulations that devalue sucrose reinforcement: change in appetite by pre-feeding. After training, BL performance of mice (*N* = 12) was recorded. Overnight animals were pre-exposed to sweet pellets *ad libitum*, and the following day, the test session started.

**Figure 7:**
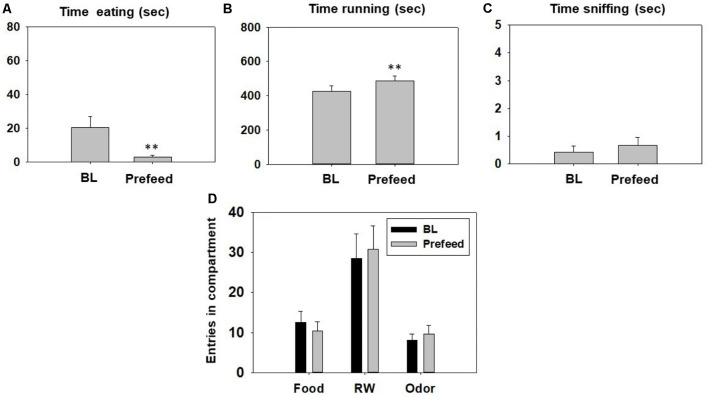
Effect of pre-feeding on time eating **(A)**, time running **(B)**, time sniffing **(C)**, entries into compartments **(D)**, and in the T-maze task assessed during 15 min. Data were analyzed using the Student’s *t*-test for dependent samples. Bars represent mean ± SEM of accumulated seconds or number of entries. **p < 0.01 significantly different from BL condition.

The Student’s *t*-test for dependent samples showed a significant increase in time running in the RW (*t*_(11)_ = −3.87, *p* < 0.01), and a decrease in time eating sucrose pellets (*t*_(11)_ = 2.95, *p* < 0.01) in the pre-feed condition compared with the control condition (Figures 7A,B). However, there was no significant effect of pre-feeding on time spent sniffing the neutral odor (*t*_(11)_ = −0.82, *p* = 0.42; Figure 7C). The *t*-tests revealed no statistical differences in number of entries into the food compartment (*t*_(11)_ = 1.51, *p* = 0.15), entries into the RW compartment (*t* = −1.02, *p* = 0.32) and entries into the odor compartment (*t*_(11)_ = −1.42, *p* = 0.18; Figure 7D).

### Experiment 9

Effect of pharmacological and food manipulations on amount of sucrose consumption, or locomotion in the T-maze. Data on pellets consumed (in milligrams) or crosses between compartments in the T-maze from experiments 5, 6, 7, and 8 were analyzed comparing each experimental condition with its respective control condition (vehicle or BL).

The Student’s *t*-test for dependent samples showed significant differences in the number of pellets consumed when animals received fluoxetine 20.0 mg/kg in comparison with their vehicle condition (*t*_(9)_ = −3.13, *p* < 0.01). However, the *t*-test comparison of vehicle-vehicle with TBZ-vehicle did not show a significant effect (*t*_(7)_ = −0.67, *p* = 0.52), and a separate *t*-test between vehicle-vehicle and TBZ-fluoxetine 20.0 mg/kg was not significant either (*t*_(7)_ = 0.88, *p* = 0.40). There were significant differences in the number of pellets consumed when animals were pre-fed (*t*_(11)_ = 2.62, *p* < 0.05), or had access to bitter food (*t*_(5)_ = 3.43, *p* < 0.05) compared to their respective controls (Table 1). The Student’s *t*-test for dependent samples showed significant differences in spontaneous locomotion in the T-maze when mice received fluoxetine 20.0 mg/kg in comparison with the vehicle condition (*t*_(9)_ = 2.53, *p* < 0.05). However, the *t*-test comparison of vehicle-vehicle with TBZ-vehicle did not show a significant effect (*t*_(7)_ = −3.11, *p* = 0.35) although a separate *t*-test between vehicle-vehicle and TBZ-fluoxetine 20.0 mg/kg was significant (*t*_(7)_ = 2.83, *p* < 0.05). Finally, there were no significant differences in spontaneous locomotion in the T-maze when animals were pre-feed (*t*_(11)_ = −0.43, *p* = 0.67) or had access to bitter pellets (*t*_(5)_ = −1.01, *p* = 0.35; data are shown in Table 2).

**Table 1:** Effect of tetrabenazine (TBZ) and fluoxetine alone or in combination, and food manipulations on pellets intake.

Pellet intake (mg in 15 min)		
	Control condition	Experimental condition
Exp. 5. Fluoxetine	112.5 ± 40.4	306.0 ± 74.9**
Exp. 6. TBZ	129.4 ± 54.1	196.9 ± 64.7
Exp. 6. TBZ+ Fluoxetine	129.4 ± 54.1	61.9 ± 35.0
Exp. 7. Bitter food	345.0 ± 104.4	41.3 ± 28.7*
Exp. 8. Pre-feeding	240.0 ± 50.7	60.0 ± 15.0*

Mean (± SEM) of milligrams consumed during the T-maze test. Data were analyzed using a series of Student’s *t*-test for dependent samples. *p < 0.05, **p < 0.01 significantly different from its respective control condition.

**Table 2:** Effect of tetrabenazine (TBZ) and fluoxetine alone or in combination, and food manipulations on general locomotion.

Locomotion in the T-maze (crosses in 15 min)		
	Control condition	Experimental condition
Exp. 5. Fluoxetine	41.5 ± 8.8	24.5 ± 4*
Exp. 6. TBZ	19.4 ± 2.4	22.5 ± 2.3
Exp. 6. TBZ+ Fluoxetine	19.4 ± 2.4	9.1 ± 2.1*
Exp. 7. Bitter food	27.6 ± 2.8	33.0 ± 1.5
Exp. 8. Pre-feeding	49.0 ± 10.0	50.8 ± 9.7

Mean (± SEM) of total entries to the three compartments of the T-maze test. Data were analyzed using Student’s *t*-test for dependent samples in each experiment. *p < 0.05 significantly different from its respective control condition.

## Discussion

The present experiments evaluated the ability of the 5-HT uptake inhibitor fluoxetine to produce antidepressant effects on the classical rodent paradigm, the FST, and also its potential to reverse the depression-like effects of the DA depleting agent TBZ in this paradigm (Carratalá-Ros et al., 2020). We confirmed that administration of a range of doses of fluoxetine significantly increased the time mice spent climbing and swimming, consequently reducing the time animals spent immobile in the FST (Figure 1). Previous studies have shown the same pattern; fluoxetine increases active behaviors (swimming and climbing) and decreases passive ones (immobility) in different strains of mice after acute or chronic administration of this SERT blocker (Lucki et al., 2001; Dulawa et al., 2004; Sanmukhani et al., 2011; Costa et al., 2013). However, when we evaluated the ability of the highest dose of fluoxetine (20.0 mg/kg) to reverse the anergic effects produced by the DA depleting agent TBZ in the FST (Carratalá-Ros et al., 2020), we observed that fluoxetine failed to reverse the effects of TBZ. Thus, animals that received the combination of TBZ and fluoxetine significantly increased time spent immobile and decreased time spent climbing, suggesting that fluoxetine is not able to alleviate motivational deficits in swimming and climbing induced by DA depletion. TBZ was used in this study as a tool for altering behavioral activation and effort-related choice since this drug has been reported to induce depressive symptoms including fatigue in people (Frank, 2009; Chen et al., 2012) and behavioral impairments in traditional rodent depression models (Wang et al., 2010; Carratalá-Ros et al., 2020), and effort-based choice tasks (Nunes et al., 2013; Yohn et al., 2016b; López-Cruz et al., 2018; Rotolo et al., 2019). These actions are likely to be produced by DA depletion since depletion of 5-HT had no effect on effort-related decision making (Denk et al., 2005). More importantly, it has been shown that a low dose of TBZ in rats reduced striatal DA by about 75%, while this same dose reduced 5-HT and NE by about 15–30% (Pettibone et al., 1984). Moreover, TBZ is 10 times more potent at reducing striatal tissue DA than at reducing 5-HT. Similar results were shown by Tanra et al. (1995), who reported that a low dose of TBZ administered to rats produced a 57% reduction in striatal DA, whereas, with 5-HT, there were no significant reductions in frontal cortex, striatum or hippocampus, and only a 20% reduction in the hypothalamus. Fuenmayor and Vogt (1979) showed that a dose of TBZ that reduced striatal DA by 87% only reduced by 51%.

The antidepressant-like effect of fluoxetine alone in the FST cannot be explained by anxiolytic actions since the same doses of fluoxetine that had an antidepressant effect in the FST, also increased anxiety-related parameters evaluated in the DL box and EPM paradigms. Thus, mice that received the two highest doses of fluoxetine (15.0 and 20.0 mg/kg) spent significantly less time in the lit chamber of the DL box and reduced the ratio of entries in the open arms of the EPM paradigm (Figure 2). Some animal studies have shown that acute administration of doses of fluoxetine like the ones used in the present study (20.0 mg/kg) produce anxiogenic effects in rats (Greenwood et al., 2008), and mice (Kurt et al., 2000; Belzung et al., 2001). It also has been shown that null-SERT mutant mice usually display anxious behaviors (Holmes et al., 2002). Moreover, using a chemogenetic approach, although both acute- and chronic activation of dorsal raphe nucleus serotonergic neurons induce antidepressant-like responses in the FST, only acute activation induces anxiogenic-like behaviors in rats (Urban et al., 2016). It is possible that fluoxetine induced an acute anxiety response that energizes escape-oriented behaviors in a context that is already stressful. On the other hand, in the present and previous experiments, TBZ showed no anxiogenic effects at a broad range of doses (Correa et al., 2018; Carratalá-Ros et al., 2020). Interestingly, unlike fluoxetine alone, the combination of fluoxetine plus TBZ did not produce anxiety-related effects in any of the paradigms (Figure 3).

The FST provides some information about behaviors related to the maintenance of vigorous and persistent active responding (Gil and Armario, 1998; Slattery and Cryan, 2012) in order to escape a stressful and unknown situation. In addition, we decided to evaluate the effects of fluoxetine in a non-stressful habitual context; the 3-choice-T-maze-task, a rodent model that evaluates preference for vigorous physical activity vs. other more sedentary sources of reinforcement (Correa et al., 2016; López-Cruz et al., 2018; Carratalá-Ros et al., 2020). In the standard version of this paradigm, no stressor is used, and mice have been extensively in contact with the T-maze and the reinforcers (2 weeks of BL). They can freely distribute their time performing an effortful and highly preferred activity (running in a RW) or engaging in more sedentary activities (eating sucrose pellets or sniffing a fruit odor). Under basal conditions, mice spent most of their time running in the RW, some of the time eating sucrose pellets or drinking a sucrose solution in other versions of the T-maze, and very little time sniffing the neutral odor (Correa et al., 2016, 2020). In this experiment, the highest dose of fluoxetine (20.0 mg/kg) significantly decreased the time mice spent running and partially shifted their behavior, increasing time consuming sucrose pellets in comparison with the vehicle condition (Figure 4). This increase in time consuming sucrose is also seen in terms of the amount of food consumed; the highest dose of fluoxetine significantly increased intake of sucrose in milligrams (around 170%, Table 1). This pattern of effects is surprisingly similar to the pattern previously observed for TBZ alone in this paradigm (López-Cruz et al., 2018; Carratalá-Ros et al., 2020). The administration of different doses of TBZ produced a partial shift; mice spent less time running and increased time spent consuming sucrose pellets in comparison with the vehicle condition. TBZ alone also tended to increase total milligrams consumed, but the increase was milder (around 35–50%), and never reached significance (Carratalá-Ros et al., 2020). The present fluoxetine-induced increase in food consumption has not been observed in rats in operant choice procedures, and in fact, fluoxetine decreased lever pressing for palatable food as well as intake of chow (Yohn et al., 2016b). It is possible that this discrepancy is due to differences between these two species. Male rats tend to consume proportionally more food and are less active than mice in T-maze paradigms (Presby et al., 2020). Thus, it is possible that mice are more sensitive to the anergic effects of fluoxetine (Table 2), and much less to its appetite suppressant effects. Moreover, although previous clinical studies have shown how this SERT blocker decreases appetite (Silverstone, 1992; Michelson et al., 1999), a recent metanalysis study (Serralde-Zúñiga et al., 2019) comparing different categories of anti-obesity drugs indicates that, unlike other drugs, fluoxetine treatment does not conclusively decrease weight, but there is a clear increase in the risk for drowsiness and fatigue. In addition, unlike TBZ alone, mice treated with 20.0 mg/kg of fluoxetine showed avoidance for the RW in general; not only they spent significantly less time running, in addition, they also reduced the total number of entries into the RW compartment, an effect that was not observed for the other compartments. This is a pattern of effects that is similar to that produced by introducing aversive conditions in the T-maze (Carratalá-Ros et al., 2020). Placing an intense light over the RW, which is known to induce anxiogenic effects in the DL, shifts behavior from RW to food consumption, but animals also show place avoidance for the RW compared to BL (Carratalá-Ros et al., 2020). Thus, although fluoxetine may have anxiolytic effects in other contexts, it is possible that in the context of the RW T-maze, fluoxetine at this dose (Figure 2) is having anxiogenic effects, or producing an aversion to the RW. However, those results are different from the pattern observed for TBZ, which did not change any of these parameters; mice still highly preferred to spend time in the compartment with the active reinforcer (the RW compartment) after TBZ administration, although they did not run as much (Carratalá-Ros et al., 2020).

In the next experiment, different doses of fluoxetine were unable to reverse the partial shift from RW towards food induced by TBZ (Figure 5). However, fluoxetine at all doses suppressed the TBZ-induced increase in time consuming sucrose, making the combined administration of the two drugs no different from control conditions. In previous studies in rats, fluoxetine consistently failed to reverse the lever pressing suppression induced by TBZ (Yohn et al., 2016b). But when animals were tested on fluoxetine alone, they did not shift their behavior from high effort/highly palatable food to free chow available in the operant chamber, as is typical for TBZ (Yohn et al., 2016c). In fact, in these experiments, fluoxetine also reduced chow consumption (Yohn et al., 2016c). Thus, it seems that fluoxetine in combination with TBZ produces both; anergic effects and appetite suppressant effects in rats and mice. For that reason, we wanted to evaluate the effects of conditions related to appetite and food reinforcement on the T-maze choice task, to compare those actions with the effects of fluoxetine plus TBZ (Figures 6, 7). We devalued sucrose reinforcement by pre-feeding the mice with the same type of sucrose pellets the night before the test, or by adding quinine to the sucrose pellets in order to make them bitter. Both manipulations produced a significant shift in behavior substantially different from those produced by fluoxetine or TBZ; devaluation of sucrose reinforcement reduced almost to zero the time mice spent consuming sucrose pellets in comparison with the BL condition, but significantly increased time running, which was not observed in the pharmacological interaction experiment (Figure 5). Moreover, pre-feeding or making the pellets bitter led to a robust and significant reduction of milligrams consumed, which did not occur either with TBZ alone, and did not reach significance with TBZ plus fluoxetine (Table 1). Previous results have shown that the effects of DA antagonists and TBZ do not resemble the effects of reinforcer devaluation on effort-based choice performance in rats (Randall et al., 2012, 2014) and mice (Yang et al., 2020). Thus, the present results strongly confirm that the actions of TBZ on these paradigms should not be interpreted as an effect on the primary or unconditioned reinforcing value of food, or a food “reward” effect, but rather on the tendency to engage in activities that require exertion of physical effort.

The disruptive effects on time running produced by fluoxetine or fluoxetine in combination with TBZ in the T-maze could be explained by general effects on locomotor activity. Thus, in the anxiety paradigms, fluoxetine (15.0 and 20.0 mg/kg), or fluoxetine (20.0 mg/kg) plus TBZ reduced locomotion, as measured by the total number of entries into compartments and crosses between arms (Figures 2B, 3B,E). Moreover, in all the experiments, the number of overall crosses between compartments in the T-maze was only significantly reduced after fluoxetine (20.0 mg/kg) or fluoxetine plus TBZ (Table 2 and Figure 5D). Previous studies have reported that SERT blockade or deletion in mice reduced locomotion (Holmes et al., 2002; Sanders et al., 2007), and fluoxetine in rats that had received TBZ showed further decreased locomotor activity compared with administration of TBZ alone (Podurgiel et al., 2015).

The inability of fluoxetine to alleviate motivational impairments induced by a DA depleting agent, and to induce psychomotor deficits, could be due to the actions of fluoxetine on the mesolimbic DA system (Cameron and Williams, 1995; Daw et al., 2002). Nacb core is a central nucleus for the regulation of effort base-decision making (Cameron and Williams, 1995; Daw et al., 2002). Fluoxetine alone decreased DA levels in Nacb core as measure by microdyalisis (Yohn et al., 2016c), and co-administration of TBZ and fluoxetine further decreased DA tissue levels in the rat ventrolateral neostriatum compared with TBZ alone (Podurgiel et al., 2015). Nacb receives projections from dorsal raphe nuclei 5-HT neurons, and the stimulation of these neurons decreases DA mesolimbic activity (Ichikawa and Meltzer, 1995; di Mascio et al., 1998; di Giovanni et al., 2000; di Matteo et al., 2008; Browne et al., 2019). Thus, the combination of fluoxetine with TBZ, which also has been demonstrated to reduce DA tissue content and release (Nunes et al., 2013; Podurgiel et al., 2015; López-Cruz et al., 2018), suggests that these impairments in behavioral activation assessed in the 3-choice-T-maze task and in the FST could be produced by an overall reduction of mesolimbic DA activity.

In summary, acute fluoxetine energized behavior under stressful conditions in the FST but did not reverse the anergic effects produced by TBZ in either the FST or the 3-choice-T-maze task. Although acute and chronic fluoxetine had the same impairing effects in an effort-choice test in rats (Yohn et al., 2016b,c,e), because fluoxetine is administered chronically as an antidepressant, further studies should evaluate if this pattern of administration of fluoxetine can improve the impairing effects of TBZ in the 3-choice-T-maze task.

Although several clinical reports suggest that fluoxetine is relatively ineffective for treating psychomotor and motivational symptoms seen in depression and, in fact, it can exacerbate or induce these effects (Katz et al., 2004; Nutt et al., 2007; Padala et al., 2012; Rothschild et al., 2014), it should be pointed out that drugs that facilitate 5-HT can treat other aspects of depression such as mood dysfunction, rumination, and cognitive arousal (Carr and Lucki, 2011; Bell et al., 2013) and, as seen in the present study as well as previous studies, fluoxetine administered alone produces an effective response in classical antidepressant tests such as the FST (Armario et al., 1988; Cryan et al., 2005a, b; Jang et al., 2009).

Thus, the present studies are consistent with the idea that not all antidepressants are adequate for the treatment of psychomotor slowing and anergia symptoms commonly seen in depression. This idea of different therapeutic drugs having positive actions for some symptoms, but no effect or even negative actions on other symptoms, is consistent with the research domain criterion (RDoC) approach that highlights the importance of describing the neural circuits that mediate specific symptoms in psychopathology, and not simply the traditional diagnostic categories (Cuthbert and Insel, 2013).

## Data Availability Statement

The raw data supporting the conclusions of this article will be made available by the authors, without undue reservation.

## Ethics Statement

The animal study was reviewed and approved by the Institutional Animal Care and Use committee of Universitat Jaume I. Protocol authorization number by GV: 2020/VSC/PEA/0050.

## Author Contributions

CC-R: performed experiments, analysis of data, and writing of manuscript. LL-C: performed experiments and analysis of data. AM-V and RO-G: performed different experiments. JS: design of experiments and writing of manuscript. MC: design of experiments, supervision of experimental phase, analysis of data, and writing of manuscript. All authors contributed to the article and approved the submitted version.

## Conflict of Interest

The authors declare that the research was conducted in the absence of any commercial or financial relationships that could be construed as a potential conflict of interest.
